# Potential Health Benefits From Downhill Skiing

**DOI:** 10.3389/fphys.2018.01924

**Published:** 2019-01-14

**Authors:** Martin Burtscher, Peter A. Federolf, Werner Nachbauer, Martin Kopp

**Affiliations:** Department of Sport Science, University of Innsbruck, Innsbruck, Austria

**Keywords:** alpine skiing, cardiovascular, neurophysiology, biomechanics, psycho-social

## Abstract

**Objectives:** Downhill skiing represents one of the most popular winter sports worldwide. Whereas a plethora of studies dealt with the risk of injury and death associated with downhill skiing, data on its favorable health effects are scarce. A more comprehensive overview on such effects might emerge from a multidisciplinary perspective.

**Methods:** A literature search has been performed to identify original articles on downhill/alpine skiing interventions or questionnaire-based evaluation of skiing effects and the assessment of health effects (cardiorespiratory, neurophysiological, musculoskeletal, psycho-social).

**Results and Discussion:** A total of 21 original articles dealing with potentially favorable health effects resulting from downhill skiing were included in this review. Results indicate that downhill skiing, especially when performed on a regular basis, may contribute to healthy aging by its association with a healthier life style including higher levels of physical activity. Several other mechanisms suggest further favorable health effects of downhill skiing in response to specific challenges and adaptations in the musculo-skeletal and postural control systems, to exposures to cold temperatures and intermittent hypoxia, and/or emotional and social benefits from outdoor recreation. However, reliable data corroborating these mechanisms is scarce.

## Introduction

Downhill skiing represents one of the most popular winter sports worldwide. Over 2,000 downhill ski areas are spread across 67 countries with an estimated 400 million skier days annually (Vanat, 2018). Whereas, a majority of research studies so far dealt with the risk of traumatic and non-traumatic events during downhill skiing (Hagel, 2005; Burtscher and Ponchia, 2010), only a few focussed on beneficial health aspects (Müller et al., 2011b). However, in those millions of people practicing downhill skiing during the winter season, skiing becomes part of regular physical activity. Related benefits should include at least two aspects: (1) higher exercise levels during leisure-time are known to be generally associated with a healthier life style (Mensink et al., 1997) and (2) skiing may contribute to reach well-accepted minimal recommendations for physical activity (150 min of moderate or 75 min of vigorous activity per week; Thornton et al., 2016). Regular physical activity is closely associated with the individual performance level, which in turn is inversely related to mortality. For instance, the mortality risk was shown to be about 50% lower in subjects with an exercise capacity of 7–10 metabolic equivalents (METs; 1 MET = 3.5 ml O_2_/min/kg) compared to those not achieving five METs (Kokkinos et al., 2008). Performance level was even a more important predictor of mortality than cardiovascular risk factors like well-established risk factors like dyslipidemia, systemic hypertension, diabetes, or smoking (Kokkinos, 2008, Myers et al., 2002).

Moreover, repeated exposures to environmental stresses like high altitude (hypoxia) and cold might provoke adaptations and thus contribute to favorable effects (Burtscher and Ruedl, 2015; Burtscher et al., 2018). Beneficial health consequences may at least partly be mediated by diminishing the high prevalence of major cardiovascular risk factors in the general population, by improving individual cardiorespiratory fitness and motor abilities, as well as psychosocial wellbeing. Although such favorable effects are very likely to result from downhill skiing, only few studies have addressed this issue and there is no review summarizing the results of existing studies. Therefore, the aim of the present review was to highlight potential health effects from downhill skiing primarily focussing on cardiopulmonary and metabolic, neurophysiological, biomechanical, and psycho-social aspects.

## Methods

**Figure 1:**
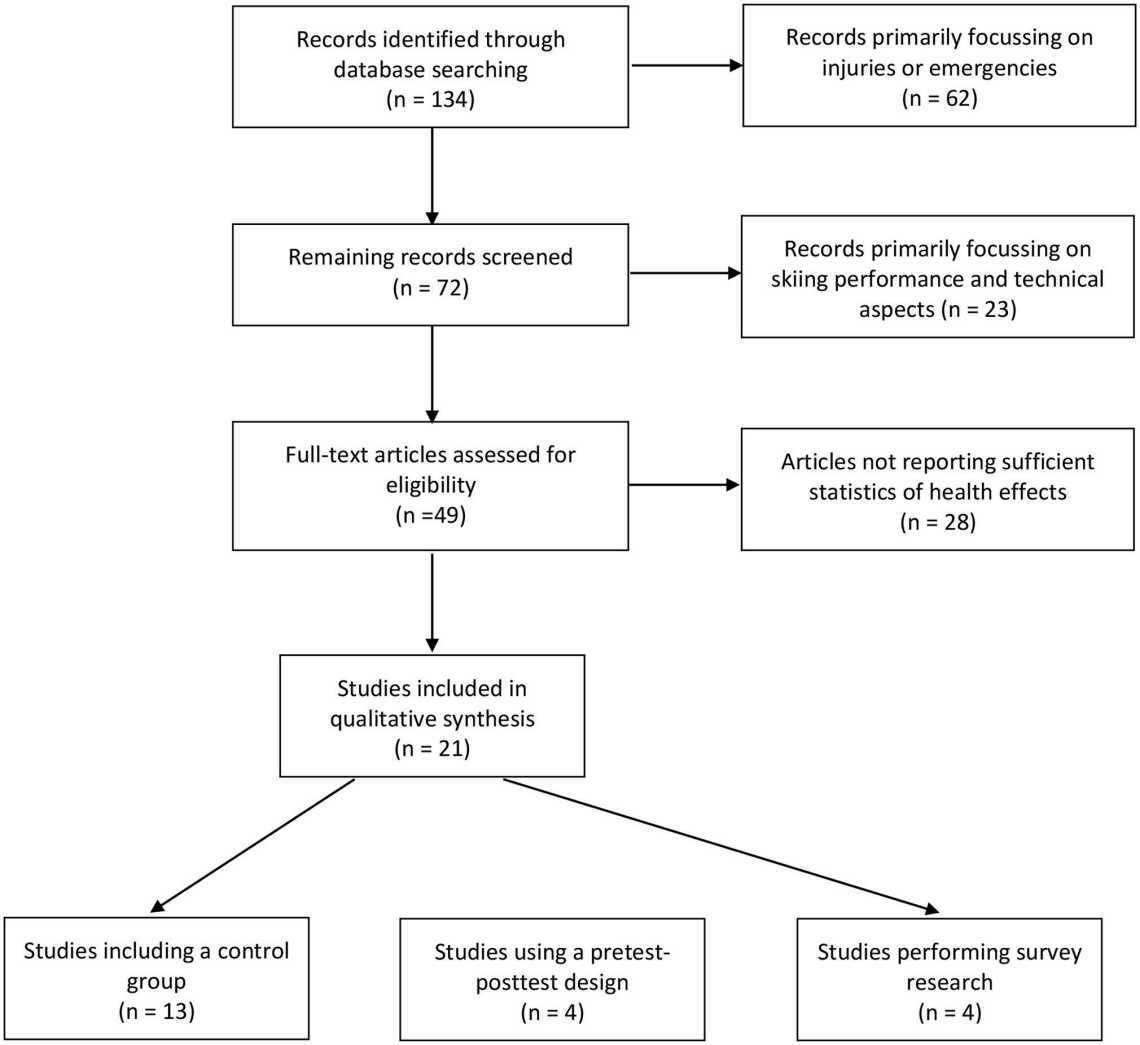
Flow diagram for search and selection of the included studies.

Titles, abstracts and relevant full-text articles have been verified by the authors using the following specific inclusion and exclusion criteria: (1) inclusion of original articles on downhill/alpine skiing interventions or questionnaire-based evaluation of skiing effects; (2) assessment of health effects (cardiorespiratory, neurophysiological, musculoskeletal, psycho-social); (3) providing statistical significance for reported effects; (3) exclusion of studies primarily dealing with skiing injuries, skiing performance or technical aspects (Figure 1). The search for papers was performed within the following databases until June 2018: Pubmed/Medline, Web of Science, Science direct, Scopus, Sport Discus. The following keywords (only in English) were used: skiing (downhill or alpine), health (heart, vasculature, respiration, lung, autonomic control, muscle, bone, tendon, motor control, endurance, strength, balance, psychological, social). Reference lists of articles were also reviewed to ensure relevant studies were included.

## Results

A total of 21 original articles dealing with potentially favorable health effects resulting from downhill skiing were included in this review (Table 1). Thirteen articles included a control group, four report effects using a pretest-posttest design, and four report findings from survey research. Data do not allow to calculate effect sizes thus only *p*-values are shown for presented effects. Results have to be interpreted with caution because a large part of articles is based on the same study population (Salzburg Skiing for the Elderly Study). Nevertheless, the selected articles represent the currently available evidence for health effects related to downhill skiing. The spectrum of potentially beneficial effects primarily covers cardiopulmonary and metabolic, neurophysiological, biomechanical, and psycho-social aspects which will be discussed below.

**Table 1:** Characteristics of studies included for review.

**References**	**Characteristics *n* total (males, females), mean age, training status**	**Intervention/Design intervention type, duration survey, (no) control group**	**Beneficial effects**	***P*-value**
Alvarez-San Emeterio et al., 2011	39 (20 m, 19f), 13–16 yrs, highly trained	Adolescent elite alpine skiers 2 yrs, control group	Bone mineral density (lumbar spine)**↑**	<0.05
			Muscle mass**↑**	<0.05
Burtscher et al., 2013	1259 (971 m, 288f), 48 yrs	Long-tern alpine skiing questionnaire	Healthy life-style**↑**	<0.05
			Hypercholesterolemia**↓**	<0.05
			Systemic hypertension**↓**	<0.05
			Memory complaints**↓**	<0.05
Dela et al., 2011	42 (22 m, 20f), 67 yrs, moderately trained	Alpine skiing, 12 wks control group	Body fat**↓**	<0.05^§^
			Insulin resistance**↓**	<0.05^§^
			Leg muscle strength**↑**	<0.05
Finkenzeller et al., 2011a	42 (22 m, 20f), 67 yrs, moderately trained	Alpine skiing, 12 wks control group	Subjective strength**↑**	<0.05
			Relationship to “friends and relatives”**↑**	<0.05
Finkenzeller et al., 2011b	42 (22 m, 20f), 67 yrs, moderately trained	Alpine skiing, 12 wks control group	Psycho-physiological reactivity Heart rate variability Skin conductance level	n.s.
Flueck et al., 2011	20 (11 m, 9f), 67 yrs, moderately trained	Alpine skiing, 12 wks no controls	MCSA (m. vastus lat.)**↑**	<0.05^§^
			Fast type fibers**↑**	0.08^§^
			Slow type fibers**↑**	<0.05^§^
Fromel et al., 2017	1278 (475 m + 803f), 17 yrs	Questionnaire (oudoor activities + alpine skiing; pedometer use)	Regular physical activity**↑**	<0.001
			Well-being**↑**	<0.001
Gao and Abeysekera, 2004	70 (49 m, 21f)	Questionnaire (participation in winter sports)	Incidence of falls**↑**	<0.001
Lauber et al., 2011	42 (22 m, 20f), 67 yrs, moderately trained	Alpine skiing, 12 wks control group	Spinal reflex activity**↑**	<0.05
			Postural sway**↓**	<0.05
Mladenović et al., 2015	54 children, 7.5 yrs	Skiing learning no control	Motor ability**↓** (e.g., coordination, strength, balance)	<0.05
Müller et al., 2011a	42 (22 m, 20f), 67 yrs, moderately trained	Alpine skiing, 12 wks control group	Aerobic capacity**↑**	0.01
			Leg muscle power**↑**	0.03
			Leg muscle strength**↑**	0.04
Narici et al., 2011	42 (22 m, 20f), 67 yrs, moderately trained	Alpine skiing, 12 wks control group	Muscle thickness**↑** (m. vastus lat.)	<0.001^§^
			Leg muscle strength**↑**	<0.001^§^
Niederseer et al., 2011	42 (22 m, 20f), 67 yrs, moderately trained	Alpine skiing, 12 wks control group	Body fat**↓**	<0.001
			Aerobic capacity**↑**	<0.01
Niederseer et al., 2016	42 (22 m, 20f), 67 yrs, moderately trained	Alpine skiing, 12 wks control group	Endothelial Progenitor cells**↑**	<0.001
			Peripheral arterial tone**↓**	<0.05
			Homocysteine**↓**	<0.05
Nikander et al., 2008	23 (m), 24 yrs, highly trained	Elite mogul and slalom skiers control group	Areal bone mineral density**↑** (lumbar spine, femoral neck)	<0.05
			Bone mineral content**↑** (tibial shaft, distal tibia)	<0.05
Pötzelsberger et al., 2015a	27 (17 m, 10f), 70 ± 5 yrs 2 yrs, after total knee arthroplasty	Intervention group: skiing control group	Strength**↑**	<0.05
			Asymmetry in walking**↓**	<0.05
Schipilow et al., 2013	47 (22 m, 25f), 23 yrs, highly trained	Elite alpine skiers control group	Distal tibia: trabecular bone mineral density**↑**	<0.05f
			Total cross-sectional area**↑**	<0.05f
			Failure load**↑**	<0.05f
Seynnes et al., 2011	42 (22 m, 20f), 67 yrs, moderately trained	Alpine skiing, 12 wks control group	Patellar tendon stiffness**↑**	<0.05
Sievänen et al., 2015	26 (18 m, 8f), 23 yrs, highly trained	Elite alpine skiers control group	Bone mineral density**↑** (total body, lumbar spine, femoral neck)	<0.05
van Ginkel et al., 2015	19 (m + f), 67 yrs, moderately trained	Alpine skiing, 12 wks no controls	Capillary density**↑** (m. vastus lat.)	<0.05^§^
Wojtyczek et al., 2014	78 (54 m, 28f), 20–22 yrs	7 day ski camp no controls	Balance on unstable board**↑**	<0.001

*MCSA, muscle cross sectional area; P-values are reported for between group changes*,

§ *indicates only within group changes. m, males; f, females*.

## Discussion

### Cardiopulmonary and Metabolic Considerations

#### Description of Specific Demands/Conditions of Downhill Skiing

Downhill skiing represents an outdoor sports activity, which is typically performed during the winter season on snow-covered slopes of mountainous areas. Usual ambient conditions are characterized by rather cold temperatures and moderate altitudes (1,500–2,500 m). Skiers use ski lifts and cable cars for the ascent, which is followed by downhill turns counteracting gravity by means of muscle power (Burtscher et al., 2013). Muscle power is primarily generated by eccentric and isometric contractions. Energy supply occurs anaerobically as well as aerobically thereby provoking intensity-dependent responses of the cardiopulmonary system (Turnbull et al., 2009; Ferguson, 2010; Burtscher et al., 2013). Challenges of those systems depend mainly on the skiing velocity, the radius of turns, steepness of the terrain, snow and ambient conditions, skiing equipment, individual skiing skills, fitness, and health status. Thus, the reported large variability of cardiopulmonary and metabolic responses to downhill skiing is not surprising (Burtscher et al., 2005; Stöggl et al., 2016). In general, the skiing-related stimuli may be intense enough to generate beneficial adaptations in subjects of both sexes and a broad range of age (Burtscher et al., 2005; Niederseer et al., 2011). Stöggl and colleagues recently compared metabolic and cardiopulmonary responses of middle aged healthy males and females when downhill skiing, cross country skiing or indoor cycling (Stöggl et al., 2016). The percentages of maximal heart rate and maximal oxygen uptake were 87 and 74% during downhill skiing, 88 and 89% during cross country skiing, and 82 and 82% during indoor cycling. Blood lactate levels did not differ between groups. The authors calculated that about 2.5 h of downhill skiing (including passive ascents) would be necessary to elicit the same energy expenditure as during 1 hour of cross-country skiing or indoor cycling (Stöggl et al., 2016). Similar results were demonstrated by our study group when comparing downhill skiing and mountain hiking (Burtscher et al., 2005). Whereas, metabolic and cardiopulmonary responses differed significantly depending on the exercise intensity (moderate vs. intense) no essential differences were seen between downhill skiing and mountain hiking (Burtscher et al., 2005). Taken together, when compared to endurance type exercise, downhill skiing means primarily eccentric, and isometric interval exercise evoking similar cardiopulmonary responses. However, due to the relatively short duration of the individual downhill runs (about 1 to 5 min) several repetitions are needed for similar energy expenditure and increases of cardiopulmonary fitness. Both cold temperatures and moderate altitude (hypoxia) may represent additional stimuli contributing to beneficial effects of downhill skiing (Burtscher and Ruedl, 2015; Ihsan et al., 2015). Moreover, downhill skiing may also contribute to a higher level of leisure-time activity which is generally associated with a healthier life style (Burtscher et al., 2013).

### Specific Adaptations and Related Potential Health Effects

Based on physiological characteristics mentioned above, downhill skiing related training stimuli will evoke comparable metabolic and cardiorespiratory adaptations and health benefits as known from interval type endurance training. Especially intense skiing intervals may cause similar effects as sprint interval training, e.g., 30-s intervals, which have been suggested to be an equally effective alternative to continuous endurance training but with a reduced volume of activity (Gist et al., 2014). In addition, short high intensity intervals are well tolerated even by subjects suffering from chronic diseases like coronary artery disease (Fleg, 2016). Rather moderate skiing intensity of about 2.5 h seems to be necessary to elicit a similar energy expenditure as 1 h of endurance training, e.g., cross country skiing or indoor cycling (Stöggl et al., 2016). Thus, downhill skiing only once a week can contribute significantly to meet the generally recommended physical activity guidelines of 150 min moderate-to-vigorous physical activity per week which represents an integral component to improve or maintain cardiorespiratory fitness and of the prevention and treatment of chronic disease (Thornton et al., 2016). Nonetheless, it is recalled that downhill skiing is performed in the field, often under extremely varying conditions. Thus, individual dosing of exercise intensity is not easy and beside injuries due to falling cardiopulmonary adverse events may occur especially in subjects with low cardiorespiratory fitness and pre-existing diseases (Burtscher et al., 1993; Burtscher and Ponchia, 2010). Cardiorespiratory fitness (determined by measuring aerobic exercise capacity, VO_2_max) means the ability to transport adequate amount of oxygen by the respiratory and circulatory systems from the environmental air to the working muscles and to use the delivered oxygen efficiently by the mitochondria of skeletal muscles (Burtscher, 2013). Beside some cardiac adaptation, downhill skiing may contribute to improved VO_2_max especially by the increase in capillarity of the mainly recruited muscle groups (van Ginkel et al., 2015). There is also a well-known negative relationship between cardiorespiratory fitness and the manifestation of cardiovascular risk factors. We recently demonstrated that long-term skiing was associated with more favorable life-style characteristics and health status compared to the general population (Burtscher et al., 2013). We found a significant “dose dependent” effect of downhill skiing on self-reported cardiovascular risk factors, i.e., hypercholesterolemia, systemic hypertension, and diabetes, and memory deficits as well. Another study conducted with elderly subjects showed improved exercise capacity (cardiorespiratory fitness, VO_2_max), a decrease in body fat mass and improved glucose tolerance after 12 weeks of guided skiing compared to controls (Dela et al., 2011; Niederseer et al., 2011). In that study however, no changes were seen regarding systemic blood pressure, blood lipids, and everyday physical activity. The contribution of skiing to the improvement of glucose homeostasis is promising especially when considering the epidemic dimension regarding the prevalence of diabetes mellitus type 2 and related cardiovascular and cerebrovascular diseases (Dela et al., 2011; Bhupathiraju and Hu, 2016). Moreover, increased endothelial progenitor cells and reduced peripheral arterial tone were reported after 12 weeks of downhill skiing indicative for the preventive potential of skiing on atherogenesis (Niederseer et al., 2016). The specific effectiveness of downhill skiing on the development of cardiovascular risk factors is supported by the evidence that eccentric type of exercise may elicit beneficial effects on lipid concentrations and glucose tolerance (Drexel et al., 2008). It seems reasonable to assume that downhill skiing may not only favor healthy aging but also supports maintaining a high individual fitness level as it is known for other popular winter sports like cross-country skiing (Nikolaidis and Knechtle, 2018; Nikolaidis et al., 2018).

The intermittent hypoxia occurring during ascents and downhill runs may contribute to beneficial health effects of skiing by improving glycemic control, blood lipid profile, and/or exercise tolerance (Lee et al., 2003; Schobersberger et al., 2003; Burtscher et al., 2004). Exercise combined with cold exposure was shown to stimulate mitochondrial biogenesis more than exercise alone, representing another potential benefit of downhill skiing (Ihsan et al., 2015). Again, many of these effects are not specific for downhill skiing and may also be induced by other types of exercise but enthusiastic skiers would probably not replace all skiing by alternative exercises.

### Recommendations for Optimal Generation of Expected Health Effects

Downhill skiing may be associated with a considerable risk of injury, e.g., due to insufficient individual fitness, pre-existing diseases, risk-taking behavior, and/or inappropriate equipment (Burtscher et al., 2008; Burtscher and Ponchia, 2010). However, due to the decreasing injury risk in downhill skiers, the risk-benefit ratio has dramatically changed during the past decades (Burtscher and Ruedl, 2015). These favorable changes are primarily attributed to the introduction of short carving skis, more rigid and comfortable ski boots, the use of protective gear like helmets, and the optimized preparation of ski slopes. It is obvious that downhill skiing can significantly contribute to regular physical activity which is well accepted to reduce morbidity and mortality, predominantly those arising from cardiovascular and metabolic diseases (Thompson et al., 2003). From a cardiovascular point of view, downhill skiing seems to be safe even for elderly subjects who are free from significant chronic diseases (Niederseer et al., 2011) but the risk of severe cardiovascular adverse events increases sharply in men over the age of 35 suffering from coronary artery disease particularly with prior myocardial infarction, and/or risk factors like arterial hypertension, hypercholesterolemia, or diabetes (Burtscher and Ponchia, 2010). However, a huge risk reduction has been shown for those performing vigorous exercises more than one time per week. These studies also demonstrated that the risk, e.g., of suffering from sudden cardiac death (SCD) during downhill skiing, was greatest on the first day at altitude when 50% of all SCDs occurred (Burtscher and Ponchia, 2010). Interestingly, sleeping at higher altitude before the first skiing day reduced the SCD risk markedly indicating some protection by short-term acclimatization (hypoxia pre-conditioning; Lo et al., 2013). Therefore, each individual skier but especially those with pre-existing cardiovascular diseases can contribute to the optimization of the risk-benefit ratio by appropriate medical therapy of risk factors, the timely development of sufficient physical fitness, sleeping the first night close to the altitude where skiing will be performed, and rest or low-intensity skiing on the first skiing day.

### Neurophysiological Aspects

#### Specific Neurophysiological Demands

Downhill skiing is a relatively complex challenge to the sensorimotor system that requires coordination of movements in an environment providing a high level of external perturbations. From a neurophysiological perspective, three aspects of the alpine skiing sport appear to be particularly interesting. First, skiing is performed on an inherently slippery, and usually inclined surface, alpine skiing thus requires specific postural control skills (Paillard, 2017). Second, as already mentioned in the previous chapter, a high fraction of eccentric and isometric muscle work is characteristic for alpine skiing. Third, alpine skiing is typically a fast motion over an uneven, rough-surfaced terrain. As a result, the movement control system is constantly exposed to large scale perturbations and vibrations.

### Effects of Alpine Skiing on Balance and Postural Control

Postural control is one of the most important skills that protect from injury and ensure mobility and quality of life. Hence, several studies on alpine skiing assessed potential effects on the postural control system. Müller and colleagues assessed balance ability in elderly volunteers (age 60–76) after a 28.5 days of guided skiing (Müller et al., 2011a). Several physiologic variables changed, but none of the dynamic postural control variables showed a change over time or in comparison to the control group (Müller et al., 2011a). In static balance assessments (quiet stance) a reduced sway area and an increased H-reflex excitability in a dynamic task were reported after training (Lauber et al., 2011). Researchers from the same group also performed a study on gait asymmetry in patients after a unilateral total knee arthroplasty (age 71 ± 5 years) and reported that 12-weeks of recreational skiing intervention with skiing two to three times per week led to significant improvements in gait symmetry (Pötzelsberger et al., 2015a). The authors argue that the skiing movement, in experienced skiers, shows a symmetric loading characteristic between the two legs (Pötzelsberger et al., 2015b) and suggest that the skiing intervention may therefore have encouraged the patients to more evenly distribute the load in gait.

High quality intervention studies in other age groups are largely missing. Two studies on young adults (students, age 20–22, novice, and intermediate skiing skills) after a 7-day skiing intervention (Wojtyczek et al., 2014) and on adolescents (age 14, novice skiers) after a 5-day intervention (Camliguney, 2013) reported improved balance skills, however, due to missing control groups these results have to be interpreted with caution. Conversely, additional balance training in physical education students with no initial skiing experience improved their skiing after a 2-week skiing intervention (Malliou et al., 2004) and in young (age 11–14) skiing athletes better balance correlated with better skiing results (Lesnik et al., 2017). In a cross-sectional study Noe and Paillard compared postural performance between regional and national level male alpine skiers (age 17–25) in eyes-open and eyes-closed conditions and with and without wearing ski boots (Noe and Paillard, 2005). Astonishingly, those skiers competing at a higher level showed inferior postural performance than the less skilled skiers, specifically in the without-ski boot condition. However, this result was not confirmed in a similar study with a much larger sample size (Mildner et al., 2007). In adolescent skiing athletes (age 11–18) Raschner and colleagues reported gender differences, with 14 to 16 year old females showing better stability and sensory scores than their male peers (Raschner et al., 2017).

A recent review and meta-analysis highlights the specificity of balance training (Kümmel et al., 2016). From this perspective, the adaptations in postural control obtained through alpine skiing may be expected to be situation specific, for example, specific to wearing a ski boot (Noé et al., 2009; Bottoni et al., 2014; Staniszewski et al., 2016). Improvements in postural stability may also relate less to laboratory tests for static or dynamic stability (Cigrovski et al., 2017, Panjan et al., 2016), but may have a stronger effect on postural control during sliding or slipping. In fact, a questionnaire study conducted in Sweden reported significantly lower number of slip falls in participants of winter sports compared to non-participants (Gao and Abeysekera, 2004). Furthermore, in ski jumpers a superior balance response to standing surface perturbations was reported compared to a control group (Mani et al., 2014).

### Neurophysiologic Adaptations in Response to Alpine Skiing

The number of studies on adaptations to a skiing intervention in specific neurophysiological functions and variables is quite limited. Previous research focused on reflex excitability, on signaling to the muscles and their coordination, on cognitive function, and a number of papers suggested neurophysiological adaptations due to exposure to vibrations in skiing. An increase in H-reflex excitability, as reported by Lauber et al. (2011), is a typical adaptation to a balance training intervention in older subjects. Similar adaptations have been reported for other interventions such as tai chi (Chen et al., 2011) or combined strength and balance exercises (Penzer et al., 2015). Conversely, in younger subjects inhibition of the H-reflex is a more common observation after balance training (Taube et al., 2007) or, for instance, a slackline intervention (Keller et al., 2012); however, to the best of our knowledge, no data on young participants is yet available for skiing interventions. Changes in reflex excitability point toward spinal and supra-spinal adaptation mechanisms (Taube, 2012, Taube et al., 2007), however, skiing specific findings are not yet available.

The high eccentric forces acting on the skier leads to adaptations not only within the muscle, but also in the neural signaling to the muscle, and in the central nervous system (Fang et al., 2004). Vogt and Hoppeler evaluated muscle coordination patterns in elite skiers and reported very high correlations between a coordination quality index and skiers' world rank in slalom (but not downhill rank; Vogt and Hoppeler, 2014). Kröll and colleagues investigated muscle activation patterns in recreational skiing and highlighted the ability of situation-dependent changes in muscle recruitment (Kröll et al., 2010) and the ability of altering muscle coordination as important mechanisms for injury prevention.

There is very little research on effects of skiing on sensing, neural signaling or cognitive function, except for one recent study by Racinais et al. (2017), who report that elite alpine skiers showed a significantly better proprioceptive acuity than a control population, but only when tested in their ski boots. The elite skiers were also able to maintain their performance during a cognitive task in a cold environment (Racinais et al., 2017).

The effect of the vibration exposure on skiing athletes has been discussed in a number of studies (Babiel et al., 1997; Nemec et al., 2001; Federolf et al., 2009; Fasel et al., 2016; Spörri et al., 2017). One acute mechanism that may play a role in the adaptation to vibrations is muscle tuning (Nigg and Wakeling, 2001; Federolf et al., 2009; Nigg et al., 2017). Of the numerous other beneficial neuromuscular adaptations in response to vibration exposure during exercising that have been discussed in the literature (Cardinale and Wakeling, 2005; Ritzmann et al., 2014), none have been proven for alpine skiing.

### Recommendations for Optimal Generation of Expected Health Effects

While the number of high-quality publications, particularly of intervention studies that include a control group, is very limited, it may be appropriate to summarize the state of current neurophysiological research on health benefits of alpine skiing in the following way. Of the various neurophysiologic adaptations, improvements in balance and motor control skills are arguably the most tangible effects that skiing can have on health. Young and novice skiers seem to exhibit the most notable changes. However, in the elderly alpine skiing may also serve as valuable exercises for preserving postural control abilities and may specifically be useful in rehabilitation, for example, when a medical intervention has disturbed the symmetry of movement patterns.

### Biomechanical Aspects

Much research dealt with biomechanical aspects of downhill skiing. Not surprising that biomechanical considerations contribute significantly to enhance safety in recreational skiing. Examples are the tibia failure studies for the binding setting (Asang, 1976) or the analysis of ACL injuries and their prevention (Freudiger and Friederich, 2000). Moreover, numerous attempts were made to boost movement technique by biomechanical movement analyses (Muller and Schwameder, 2003). Comparatively small is the biomechanical input in determining health benefits of recreational alpine skiing.

### Effects on Skeletal Muscles

Downhill skiing is characterized by repetitive loading of the leg muscles at a wide range of intensities. The loading intensity is mainly determined by skiing speed and turn radius which vary strongly depending on skill level as well as slope and snow conditions. Indications of the strong mechanical stimulus of recreational skiing were provided by measurements of knee joint extension moments up to 250 Nm and valgus moments up to 140 Nm during parallel turning (Schindelwig et al., 1999). In a skiing study for elderly (67 ± 2 years) it was shown that 28 days guided skiing over 12 weeks led to a significant increase in leg muscle strength and power (Müller et al., 2011a) as well as in muscle thickness, fascicle length and pennation angle (Flueck et al., 2011; Narici et al., 2011). Both fast and slow type fibers contributed to the increase in thickness of working muscles (Flueck et al., 2011). The muscular improvements were similar to resistant training of older people as reported in the review of Narici et al. (2004). Resistance training has demonstrated in both young (Schoenfeld, 2013) and elderly people (Csapo and Alegre, 2016) that even training at low to moderate intensities may cause substantial gains in muscle strength and size provided that a sufficient number of repetitions is performed. Considering the high number of repetitions and moderate to high intensities, alpine skiing is therefore expected to induce substantial strength gains and muscle hypertrophy in all age groups.

### Eccentric Muscle Activation

Eccentric muscle activation is a specific feature of alpine skiing. Eccentric contractions are in particular done by the quadriceps muscle during a large portion of the turns. In giant slalom turns of elite skiers, for example, the eccentric quadriceps activation was twice as long as and also stronger than the concentric activation (Berg et al., 1995). The unique aspect of eccentric muscle activation is that much greater forces are generated at lower metabolic cost compared to concentric or isometric activation (LaStayo et al., 2003). Consequently, alpine skiing provides chronically high force production and may also be appropriate for people with low cardiorespiratory fitness. In addition, the high muscle force of eccentric activation may induce tendinous adaptation which was reported for eccentric resistance training (LaStayo et al., 2003). In fact, an increase in stiffness and Young's modulus of the patellar tendon, equivalent to a rejuvenation of tendon mechanical properties, was measured for recreational skiers after a 12-week guided skiing program (Seynnes et al., 2011).

### Effects on Bones

Another specific feature of downhill skiing is its considerable impact on the bony skeleton. Several studies suggest that alpine ski racing and the linked dryland and ski training cause an increase in strength of the weight bearing bones. This was shown for elite adult skiers (Nikander et al., 2008; Schipilow et al., 2013; Sievänen et al., 2015) as well as for elite adolescent skiers aged 13 to 16 years (Alvarez-San Emeterio et al., 2011). As stimuli for the bone adaptations are discussed in particular vigorous eccentric and concentric muscle forces, high impact forces, and high-frequency and high-magnitude vibrations (Sievänen et al., 2015). Although in recreational skiing all these stimuli are substantially lower than in ski racing, the loading characteristic with high eccentric force, large number of impacts, and vibrations stays the same for recreational skiing. It would be alluring in future research to test the bone adaptation hypothesis caused by recreational skiing.

Taken together the biomechanical view on the health benefits of recreational skiing, the unique loading of the musculoskeletal system by numerous repetitions, a wide range of moderate to high eccentric force generation at low metabolic cost, impact forces of different magnitude and frequency may provoke favorable improvements to the strength of the locomotor system. Such stimuli are essential requirements for the proper development of the locomotor system of young people and to combat age-associated reductions in adults that may cause major health problems like sarcopenia or bone atrophy. Scientific studies with direct evidence of positive adaptions by recreational skiing are scarce.

### Potential Mental Health Benefits From Skiing

#### Skiing Is an Outdoor Physical Activity (PA) What Might Enhance Psychological Well-Being

As skiing takes place mainly outdoor, it seems interesting to consider potential additional psychological benefits of this activity driven by environmental factors.

The importance of environmental effects on PA behavior in general has been recognized (Sallis et al., 2016). Affective responses, and consequently adherence to PA, can be influenced by the surrounding environment. Indeed, PA in a natural environment has been shown to create larger positive effects on affective responses compared to indoor PA (Ekkekakis et al., 2000; Pretty et al., 2005; Focht, 2009; Barton and Pretty, 2010). In their meta-analysis, Barton and Pretty provided evidence for a dose-response relationship between the duration of so called green exercise and the impact on affective responses (Barton and Pretty, 2010).

According to that, research has shown that skiing was associated with pleasure, leading ultimately to a feeling of satisfaction in participants (Lee et al., 2014). In more detail, this study showed via structural equation modeling the following total effects: pleasure from skiing lead to involvement and satisfaction and skiing-experienced flow lead to satisfaction. Authors concluded, that those participating in skiing activities and socially convening around a sporting activity are likely to have positive psychological outcomes—what can be attributed to overall human well-being.

In a qualitative investigation in freeride skiers participants stated that they are motivated to engage in their sport by regularly experiencing pleasure, freedom, nature, challenge, balance, and social interactions (Frühauf et al., 2017).

Results indicating improvements of psychological well-being during and after skiing may be explained (1) by the mere exposure to nature, (2) by the effects of skiing itself, and (3) by the interaction of these variables. For the former, the psychophysiological stress recovery theory (Ulrich et al., 1991) states that the visual stimulus of nature itself may elicit positive affective responses (Berto, 2014)

### Skiing May Have the Potential to Improve Variables Closely Related to Mental Health

There is vast range of literature, indicating overall, that physical activity may have different positive mental health effects. These results seem to justify the statement of a proven worthiness for mental health from acute exercise bouts as well as from long-term physical activity but knowledge regarding skiing is still scarce.

With a repeated measure model Finkenzeller et al. could show that a guided alpine skiing intervention has marginal positive and no negative impacts on psycho-social variables in individuals 60+ years old (Finkenzeller et al., 2011b). A limiting aspect of this study that needs to be mentioned is that participants were showing high levels of life satisfaction, self-concept, health status, and low levels of depression. Nevertheless, authors detected positive effects in strength and for the satisfaction with the relationship to “friends and relatives” and concluded that a guided alpine skiing intervention lasting 12 weeks may be recommended to physically and psychologically healthy elderly persons (Finkenzeller et al., 2011b).

According to this, Amesberger et al. reported that questionnaire data assessing physical self-concept on the dimensions sportiness, endurance, and strength were positively related to the external performance criteria of endurance, concentric muscle strength, muscle power, and balance (Amesberger et al., 2011) Additionally, this longitudinal study showed that elderly individuals who are involved in a repeated measure study on physical fitness tend to relate their self-rated global fitness more and more to an endurance parameter. Another study of the same group reported enhanced well-being and no significant impact on perceived pain, exertion or knee function in elderly skilled skiers after total knee arthroplasty (TKA) following a skiing intervention (Würth et al., 2015).

Despite these inspiring data, no evidence was found that skiing might result in better cognitive performance or in enhanced psycho-physiological reactivity and recovery in individuals aged 60+ (Finkenzeller et al., 2011a). Regarding environmental conditions, alpine skiers may benefit from their sport by keeping their cognitive performance at the same level in cold temperatures (Racinais et al., 2017).

Overall, skiing seems to have some potential in terms of factors closely related to mental health. As skiing is mainly practiced in attractive mountain areas, the exposition to natural surroundings during exercising itself may provide an additional stimulus for well-being and stress-recovery. From an exercise psychological perspective, skiing may be a favorable intervention to enhance acute affective states of participants during and immediately after exercising. However, this has to be proven in controlled cross-over trials. At this stage, available research results show potential merits from skiing in terms of pleasure, flow and body image, eventually associated with life-satisfaction, and social well-being.

### Limitations

A main limitation arises from the circumstance that downhill-skiing is part of an active life-style, hardly allowing to separate its effects from those of other activities/behavior and interactions. Moreover, some bias may result from the fact that several studies presented here are part of the large “Salzburg Skiing for the Elderly Study” (Müller et al., 2011b). For example, as available data are pre-dominantly derived from group-based research, skiing-related mental health effects might be influenced by social interactions. Especially, the studies of the Salzburg group did not control for social influences; therefore, group effects, willingness to ski as well as instructor effects might have improved well-being as well, what is a severe limitation of this project (Müller et al., 2011b). Furthermore, research so far has been carried out mainly in wealthy countries, and the diversity of skiing environments such as temperature ranges and altitude variations in different countries and locations might have influenced available research results. Another limiting aspect to consider is the cost-benefit-relationship of participation in skiing for individuals and society; keeping in mind the cost of regularly skiing or skiing holidays studies might have had a selection bias by choosing participants already in a privileged socio-economic position, who are more likely healthier and happier compared to people from a lower socio-econocmic background (Hosseinpoor et al., 2012). However, the inclusion of most available studies reporting health effects associated with downhill skiing may be considered as main strength of the present review.

## Conclusion

**Figure 2:**
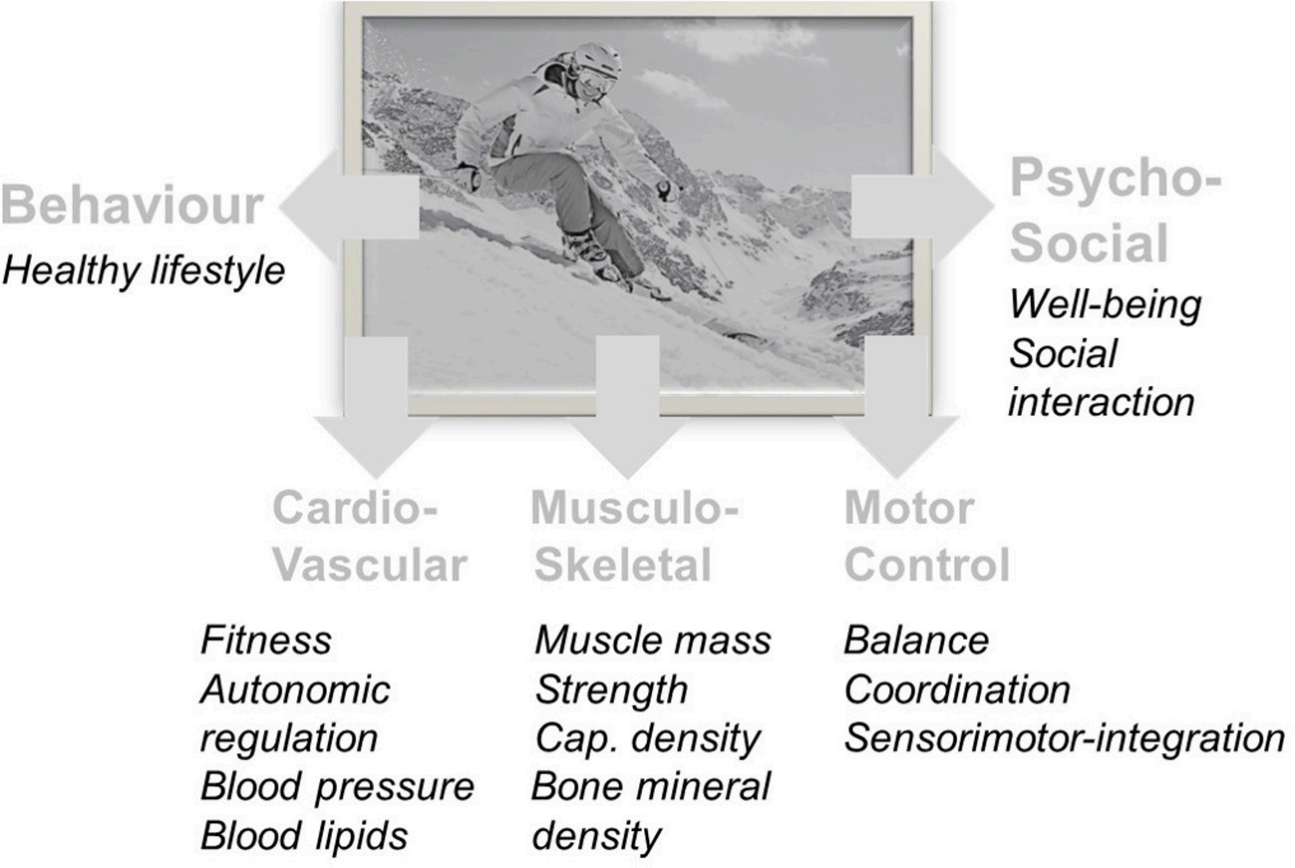
Suggested main favorable effects of downhill skiing on health.

Based on the currently available evidence it seems plausible that downhill skiing, especially when performed on a regular basis, may contribute to healthy aging by its association with a healthier life style including higher levels of physical activity. However, several other mechanisms may importantly contribute to the favorable health effects of downhill skiing, e.g., specific challenges and adaptations of the musculo-skeletal and the postural control systems, and/or exposures to cold temperatures and intermittent hypoxia, and/or emotional and social benefits from outdoor recreation. Suggested main favorable effects of downhill skiing on health are depicted in Figure 2. Nevertheless, the presented results have to be interpreted with caution and more research is necessary to confirm and extend potential health benefits by downhill skiing and finally, benefits and risks must be properly and individually weighed against each other.

## Author Contributions

All authors listed have made a substantial, direct and intellectual contribution to the work, and approved it for publication.

### Conflict of Interest Statement

The authors declare that the research was conducted in the absence of any commercial or financial relationships that could be construed as a potential conflict of interest.
